# Transcriptional Proposition for Uniquely Developed Protocorm Flowering in Three Orchid Species: Resources for Innovative Breeding

**DOI:** 10.3389/fpls.2022.942591

**Published:** 2022-06-28

**Authors:** Sagheer Ahmad, Jinliao Chen, Guizhen Chen, Jie Huang, Yang Hao, Xiaoling Shi, Yuying Liu, Song Tu, Yuzhen Zhou, Kai Zhao, Siren Lan, Zhongjian Liu, Donghui Peng

**Affiliations:** ^1^Key Laboratory of National Forestry and Grassland Administration for Orchid Conservation and Utilization at College of Landscape Architecture, Fujian Agriculture and Forestry University, Fuzhou, China; ^2^College of Life Sciences, Fujian Normal University, Fuzhou, China

**Keywords:** surprised flowering, non-vegetative, WGCNA, transcription factors, orchid development

## Abstract

During orchid seed culture, seeds germinate as protocorms, and protocorms normally develop into plant with leaves and roots. Orchids require many years of vegetative development for flowering. However, under a certain combination of growth cultures, we observed that protocorms can directly flower without leaves and roots. Therefore, we performed comparative transcriptome analysis to identify the different transcriptional regulators of two types of protocorms of *Cymbidium ensifolium*, *Cymbidium sinense*, and *Cymbidium goeringii*. Zinc finger, MYB, AP2, and bHLH were the most abundant transcription factor (TF) families in the transcriptome. Weighted gene coexpression network analysis (WGCNA) was performed to identify hub genes related to leaf and flower development. The key hubs included *SPL6*, *SVP*, *SEP2*, *KNOX1*, *AP2*, *OFP1*, *COL12*, *MYB13*, *MYB36*, *MYB59*, *bHLH086*, and *ARF7*. The hub genes were further validated through statistical tools to propose the roles of key TFs. Therefore, this study initiates to answer that why there is no leaf initiation and root development and how can protocorm bypass the vegetative phase to flower? The outcomes can direct future research on short-span flowering in orchids through protocorms.

## Introduction

Thew orchid industry provides the aesthetic nutrition to mankind. However, the most beautiful orchids spend a vegetative period of 2–3 years to reach flowering (Ahmad et al., 2021a, 2022a). Therefore, flowering time regulation is the most important topic in the orchid research. Orchids are grown through protocorms. Protocorm is the first stage of embryo development after seed germination. This term was first introduced in 1890 by Treub to describe the early stage of lycopod germination (Arditti, 1992; Yeung, 2017). Actually, orchids develop protocorm to make a symbiotic relationship with fungus to obtain nutrients for the development of shoot apical meristem (SAM). Therefore, protocorms are molecularly distinct from zygotes, as shown in *Phalaenopsis aphrodite* (Fang et al., 2016).

After fertilization, the plant embryo becomes a miniature sporophyte (Fang et al., 2016). Two distinct phases called morphogenesis and maturation occur during embryogenesis (Bentsink and Koornneef, 2008; Braybrook and Harada, 2008). Plant body components, such as functionally organized domains, apical-basal polarity, cell differentiation, and tissue specification, are organized during morphogenesis (Steeves and Sussex, 1989). However, sometimes the plants skip some phases of growth and exhibit strange phenotypes. These are abnormal changes caused by intrinsic and extrinsic factors. Genetic changes appear as strange phenotypes. Transcription factors (TFs) play important roles in the regulation of cell division planes and axis polarity (Jeong et al., 2012; Ueda and Laux, 2012).

Protocorm-like bodies (PLBs) are similar to protocorms but emerge from calluses or explants (Jones and Tisserat, 1990; Chugh et al., 2009). During PLB initiation, callus cells construct compact regions composed of meristemoids called promeristems (Lee et al., 2013). PLBs can be multiplied by cuttings, providing a quick source of obtaining orchids through clonal propagation (Yam and Arditti, 2009). The *KNOTTED1*-like homeobox (*KNOX*) genes play important role in PLB regeneration (Fang et al., 2016).

Homeobox genes play vital roles during the plant developmental process (Semiarti et al., 2014). A homeobox gene in maize codes for SAM and inflorescence meristem localized protein (Ritter et al., 2002). *KNOX* genes are recognized as TFs involving the aboveground organ development and SAM maintenance (Ritter et al., 2002; Scofield et al., 2007; Semiarti et al., 2014). KNOX1 proteins maintain a high level of cytokinin and a low level of gibberellin (GA) in SAM (Semiarti et al., 2014). In the orchids, *Dendrobium Orchid Homeobox1 (DOH1*) strongly expresses in vegetative SAM but moderately expresses in transitional SAM and floral buds (Yu et al., 2000). Its overexpression completely suppresses shoot organization in orchids. It is also an upstream regulator of *DOMADS1* (Yu et al., 2000). The *Phalaenopsis Orchid Homeobox1 (POH1*) involves the regulation of protocorm, seedling development, and the floral transition in the *in vitro* cultures of *Phalaenopsis* orchids (Semiarti et al., 2008). Abnormal leaf and shoot phenotype were observed in *Phalaenopsis amabilis* mutants with a defective C-terminal POH1 locus (Rahayu Sulistianingsih, 2013).

However, *DfKN1-4* genes from *Dactylorhiza fuchsia* expressed during floral development (Box et al., 2012; Rudall et al., 2013), suggesting the dual role of *KNOX* genes in vegetative and reproductive development. The expression timing of *KNOX* is crucial to establish a diverse range of floral morphologies (Rudall et al., 2013; Semiarti et al., 2014). Excessive functioning of KNOX proteins in various plant organs, and the misexpression of *KNOX* genes may cause the appearance of adventitious meristems from peripheral cells of explants, and the occurrence of genetic networking, leading to the growth of intact shoots that finally turn into intact plants (Semiarti et al., 2014).

The A-class MADS-box gene *AP1*/*AGL9* involves floral transition and flower organ development (Theißen, 2001). *AP1*/*SQUA*-like genes retain the conserved role of meristem identity determination (Chen et al., 2007). A number of *SEP* (*SEPALLATA*)-like E-class genes are required for floral structure formation in orchids (Salemme et al., 2013). *AP1* is a hub between flower inducing *SVP* and *SOC1*, and the determinants of floral organ identity (Honma and Goto, 2001).

R2R3-MYB TFs play important roles in flowering (Chen et al., 2022). The high expression of *FT* causes early flowering in the presence or absence of CO (CONSTANS), a B-box zinc finger TF and the main activator of *FT*. An increased expression of *MYB30* in the phloem of Arabidopsis accelerates flowering through the regulation of FT (Liu et al., 2014). The CO-independent incitement by the high expression of *MYB30* produces less *FT* expression levels as compared with those produced by CO-dependent activation. Moreover, *MYB30* dependent flowering activation is independent of FLC (Chen et al., 2022). *MYB36* regulates the transition of cells from proliferation to differentiation (Liberman et al., 2015). Ovate Family Protein1 (OFP1) regulates the growth and development of plants and together with *Arabidopsis Thaliana* Homeobox1 (ATH1), it integrates flowering time (Zhang et al., 2018). *SQUAMOSA PROMOTER BINDING-LIKE (SPL*) TFs involve flower regulation by activating MADS-box genes, such as *SOC1*, *FUL*, *LFY*, and *AP1* (Zhang et al., 2018).

With the above in mind, this study dissects the transcriptional regulation of a strange flowering that occurred in three orchid species without leaf or root formation. It opens a new era of research for rapid flowering of orchids that usually takes 2–3 years to reach flowering. TFs are the drivers of important genes, and our mining elucidates some key TFs that may serve as an input to decipher the mechanism of rapid flowering for future orchids.

## Materials and Methods

### Plant Materials and Growth Conditions

The plants from three species were obtained through protocorm development in a controlled environment. The media concentrations included: 0.5 mg L^–1^ NAA, 8.0 mg L^–1^ 6-BA, 35 g L^–1^ sugar, 1.5 g L^–1^ activated carbon, 7 g L^–1^ agar, Temperature at 26 ± 2°C, photoperiod at 12 h/day, and light intensity of 2,500–3,000 Lx. The *C. sinense* plant took about 6 months to abnormal flowering without leaves and roots, while *C. ensifolium* and *C. goeringii* both took about 90–120 days to flower.

Each species showed two types of protocorm development; the abnormal flowering plants exiting the vegetative phase and normal plants with vegetative growth. Flowering samples were obtained from abnormally developed floral buds and the leaf samples were obtained from normal plants during vegetative growth. For each species, flower and leaf samples were obtained in triplicates.

### RNA-Seq Library Preparation and Sequencing

Total RNA was extracted from 18 tissues (6 samples in 3 repeats) using the TaKaRa RNA extraction kit, and cDNA libraries were prepared. The mRNA was obtained using Oligotex mRNA Midi Kit (Qiagen, Germany). The RNA quality was assessed using a Nano-Drop spectrophotometer (Thermo Fisher Scientific, United States), followed by cDNA library preparation using the Illumina protocol (Ahmad et al., 2021a). The library products were evaluated with the Agilent 2200 TapeStation and Qubit^®^2.0 (Life Technologies, United States). The products were diluted to 10 pM for the *in situ* generation of clusters on HiSeq2500 pair-end flow cells, followed by pair-end sequencing (2 × 100 bp). Finally, transcriptome *de novo* was performed with the Trinity program using default parameters (Grabherr et al., 2011). Gene expressions were quantified using fragments per kilobase per transcript per million mapped reads (FPKM).

### Sample Correlation and Principal Component Analysis

The Pearson correlation coefficient of gene expression between samples was calculated, and the results were shown in the form of a heatmap.

Principal component analysis (PCA) is a multivariate statistical analysis method that reduces multiple variables into a few independent variables (i.e., principal components) through dimensionality reduction, while retaining as much original data information as possible. In the analysis of the transcriptome, PCA reduces the dimensionality of a large number of gene expression information contained in the sample into a few principal components that are not mutually independent, so as to compare samples, and it is convenient to find outlier samples and discriminate samples with high similarity.

The PCA analysis was performed using the princomp function in the R software, and the ggplot2 package in the R software was used to draw the graphics.

### Functional Annotation of Transcriptome Data

The assembled unigenes were mapped to public databases for annotation, such as non-redundant (NR), Kyoto Encyclopedia of Genes and Genomes (KEGG), Gene Ontology (GO), and KEGG ortholog (KO) databases, using BLASTX with a threshold E-value ≤10^–5^. The genes were also assessed on the protein family (PFAM) and UniProt databases with default parameters.

According to the GO and KEGG annotations, the differential genes were classified into functional categories and pathways. The phyper function in the R software was used for enrichment analysis. The *p* value was calculated, and corrected by FDR. The function with *Q* value ≤0.05 was regarded as significant enrichment.

### Differential Expression Analysis

We used Bowtie2 to align clean reads to genome sequences, and then used RSEM (v1.2.8) with default parameters to calculate gene expression levels for each sample. RNA-seq by expectation maximization (RSEM) is a software package^1^ for RNA-seq reads to calculate the gene and transcript isoform expression levels of *de novo* assembly (Li and Dewey, 2011; Langmead and Salzberg, 2012).

Differentially expressed genes (DEGs) were obtained using DEGseq R package (1.10.1) (Wang et al., 2010). The unigenes with a threshold *p*-value < 0.001 and the log2FC > 1 were regarded as DEGs.

### Identification of TF Families

A TF is a group of protein molecules that can specifically bind to a specific sequence upstream of the 5′ end of a gene, thereby ensuring that the target gene is expressed at a specific time and space with a specific intensity.

For animal TFs, we compared all unigenes to the AnimalTFDB 2.0 database, and obtained the corresponding database links of unigenes corresponding TF family, Ensemble gene ID, and TF family.

For plant TFs, we used getorf to detect unigene’s ORF, and then used hmmsearch to align the ORF to the TF protein domain (data from TF), and identified the unigene’s ability according to the TF family characteristics described in PlantTFDB.

### Co-expression Network Analysis

Weighted gene coexpression network analysis (WGCNA) was performed to obtain coexpression networks, as previously documented (Langfelder and Horvath, 2008; Ahmad et al., 2020, 2021a, 2022a). First, the unqualified genes were removed by using the function of goodSamplesGenes. Then, a suitable soft-threshold power was chosen based on the criteria of scale-free topology, by using a function of pickSoftThreshold. The one-gene-to-all relationship was incorporated, and the adjacency matrix was converted into topological matrix (TOM) (Yip and Horvath, 2007). The genes showing hierarchical clustering according to 1-TOM (TOM-based dissimilarity) were sorted, followed by the clustering of highly interconnected genes in the same module (Ravasz et al., 2002).

### Identification of Significant Modules and Hub TFs

From the WGCNA, the module eigengene (ME), gene significance (GS), and module membership (MM) were ascertained. MM represents the gene-module degree of correlation. For highly correlated genes, the MM should be close to 1 or -1. The hub genes represent the highly connected candidates in a module. The exportNetworkToCytoscape function was used in the WGCNA package to create edges file as input for Cytoscape (version 3.9.0). The network displayed by Cytoscape was further analyzed through CytoHubba app in the Cytoscape to filter highly connected hub genes through the degree method (Chin et al., 2014).

### Statistical Analysis to Validate Candidate TFs

One-way ANOVA was used to validate the expression of the most important TFs.

## Results

In this project, a total of 6 samples were measured using the DNBSEQ platform, and each sample produced an average of 6.53 Gb of data (Supplementary Table 1). The average alignment rate of the samples compared with the genome was 80.47%, and the average alignment rate of the compared gene set was 67.60%. The predicted new genes were 11,539. The total number of expressed genes was 33,499, of which the known genes were 23,637, and 9,862 were predicted to be new genes.

### PCA, Pearson’s Correlation, and Expression Analysis

**FIGURE 1:**
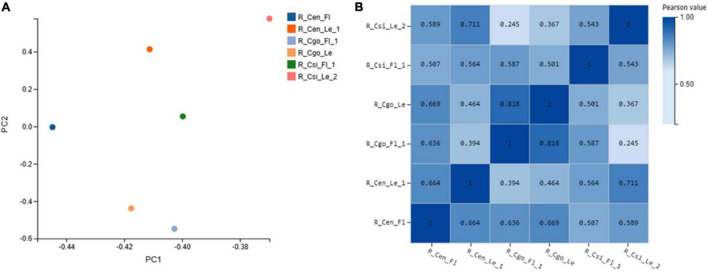
Principal component analysis (PCA) **(A)**; and Pearson’s correlation analysis **(B)**, both *X*- and *Y*-axes represent each sample. The color represents the correlation coefficient (the darker the color, the higher the correlation, and the lighter the color, the lower the correlation).

Principal component analysis represents the association among sampled tissues (Figure 1A). *C. goeringii* samples were closely associated as compared with other tissues or species.

The correlation coefficient reflects the overall gene expression between each sample. The higher the correlation coefficient, the more similar are the gene expression levels. The Pearson correlation coefficient between each two samples was calculated using the cor function in the R software (Figure 1B).

**FIGURE 2:**
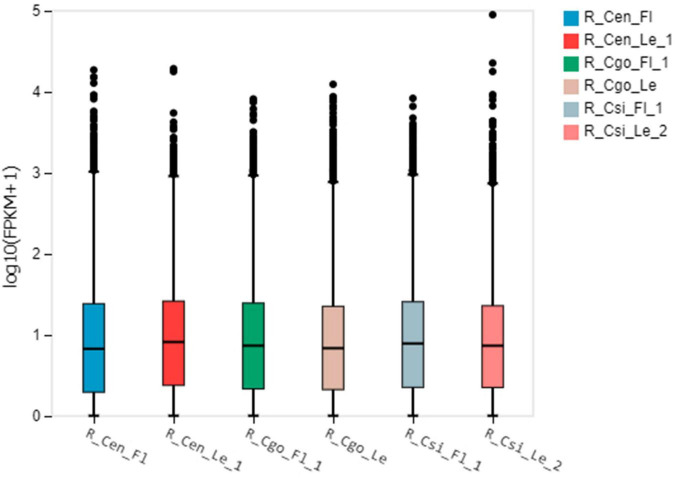
The boxplot shows the distribution of gene expression levels in each sample, and the degree of dispersion of the data distribution can be observed. The *X*-axis is the sample name, the *Y*-axis is log10(FPKM + 1), and the boxplot of each area corresponds to five statistics (from top to bottom are the upper limit, upper quartile, median, and lower quartile, respectively). Number of digits, lower bound, where upper and lower bounds do not account for outliers.

We observed the expression pattern for each tissue using the empirical cutoff values of positively expressed genes. The data are shown as boxplots (Figure 2). The boxplot distribution of FPKM values curtails the quartile and median values of DEGs among samples.

### Pairwise Comparison of DEGs

**FIGURE 3:**
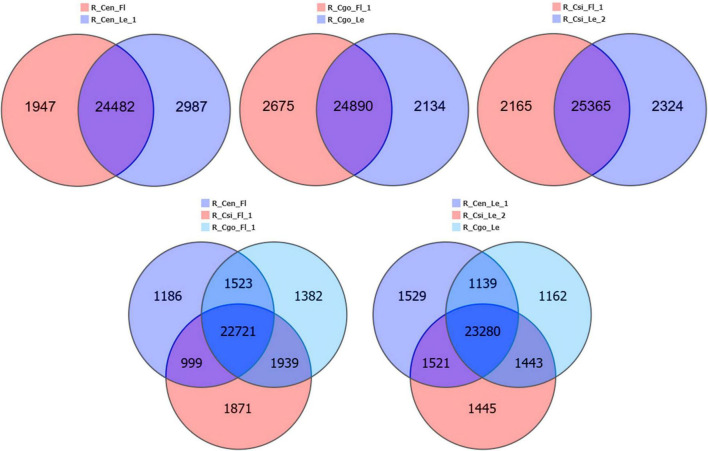
Tissue-specific and common differentially expressed genes (DEGs) among the flower and leaf samples of three orchid species.

The DEGs were compared between the flower and leaf tissues of each species and then among the flowers and leaves of three species separately (Figure 3). The maximum tissue specific expression difference can be seen in *C. ensifolium*; wherein considerable numbers of genes were expressed in the leaf as compared with flower. The ratio of common genes between flower and leaf was almost equal in all the comparisons. The comparison of flowers among the three species showed that more genes were expressed in *C. sinense* than *C. ensifolium* and *C. goeringii*. While in leaf comparison, more numbers of genes were expressed in *C. ensifolium*, although the difference was not significant with other species (Figure 3).

### GO and KEGG Enrichment

**FIGURE 4:**
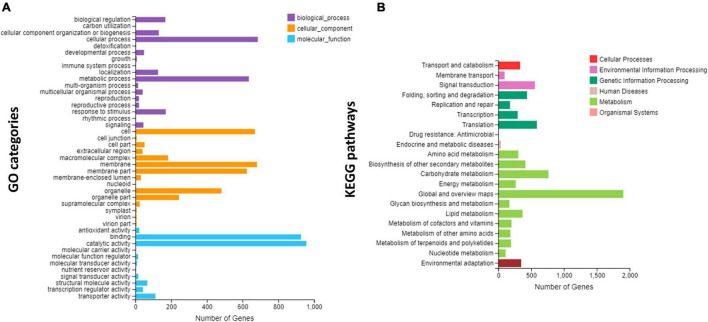
Overview of Gene Ontology (GO) and Kyoto Encyclopedia of Genes and Genomes (KEGG) enrichments; **(A)** the GO enrichment, and **(B)** the KEGG enrichment.

Gene Ontology and KEGG enrichment analyses were performed for genes expressed in three species (Figure 4). The highly enriched biological processes included cellular process and metabolic process, followed by biological regulation and response to stimulus (Figure 4A). These four biological processes are considered the most important in the regulation of unique flower development observed in the four species. The highest numbers of genes were related to cells, followed by membrane and organelle. Regarding the molecular function, the highest numbers of genes involved catalytic activity and binding (Figure 4A).

The KEGG pathway enrichment showed that most of the genes were involved in the metabolic pathways and carbohydrate metabolism, which are important pathways in the regulation of unique flowering (Figure 4B). Other important pathways included signal transduction, translation, environmental adaptation, biosynthesis of secondary metabolites, and transport (Figure 4B).

### Transcription Factors

**FIGURE 5:**
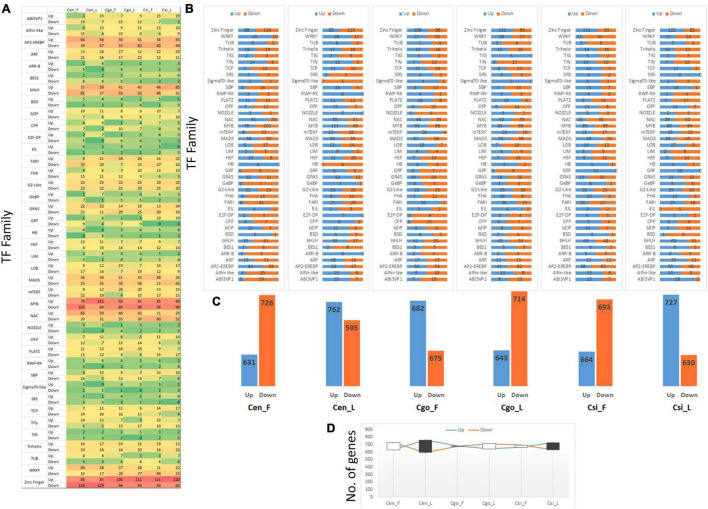
Statistics of 40 transcription factor (TF) families **(A)**, upregulation and downregulation trend of individual TF family **(B)**, difference in the upregulation and downregulation for each tissue **(C)**, and the trend of comparative upregulation and downregulation **(D)**.

A total of 2,075 TFs were identified from the transcriptome data. After removing the uncharacterized or low/null expression profiles, 1,373 TFs were selected for further analysis (Supplementary Table 2). The selected TFs were divided into 40 TF families (Figure 5A). The prominent TF families with higher number of DEGs included zinc finger, MYB, bHLH, and AP2-EREBP (Figure 5A). The most upregulated expression was shown by zinc finger TFs, showing downregulation in *C. ensifolium* and upregulation in *C. goeringii* and *C. sinense*. However, the NAC family showed upregulation in the *C. ensifolium* and *C. goeringii*, and downregulation in *C. sinense*.

A higher downregulation trend can be seen for all the families in the flowers of *C. ensifolium* as compared to other tissues or species (Figure 5B). Interestingly, the highest number of downregulated genes can also be seen in the leaf of *C. ensifolium*. The other two species showed an almost equal upregulation and downregulation trend.

Significantly opposite expression profiles between the flowers and leaves can be seen in the *C. goeringii* and *C. sinense* as compared with *C. ensifolium* (Figure 5C). A significant number of genes were upregulated in the flowers of *C. goeringii*, while a large number of genes were downregulated in the leaves. However, the trend was opposite for *C. sinense*, wherein a large number of genes were downregulated in the flowers as compared with leaves (Figure 5C).

The lowest difference between the upregulated and downregulated genes was observed in the leaves of *C. ensifolium* as compared with other tissues (Figure 5D).

### Weighted Gene Coexpression Network Analysis

**FIGURE 6:**
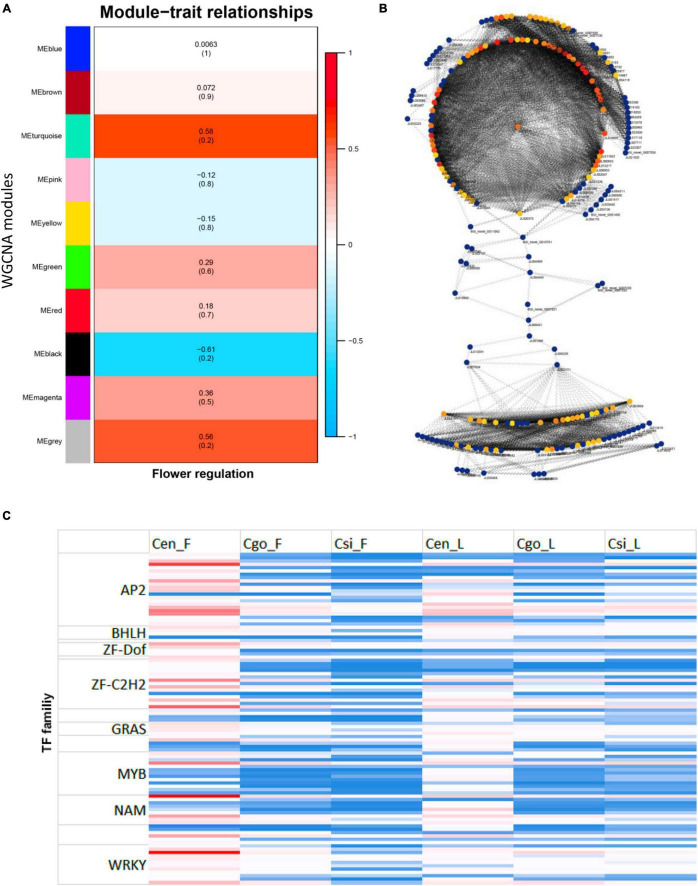
Identification of modules through the weighted gene coexpression network analysis (WGCNA) **(A)**; interaction network by modules **(B)**, the color intensity shows the level of connectivity (red for high connectivity and yellow for low connectivity); and the abundance of TF families for top 100 highly connected TFs identified from modules **(C)**.

A WGCNA was performed to identify coexpressed gene sets in the flower and leaf transcriptomes of three orchid species (Figure 6). From 1,373 TFs, a total of 10 modules were ascertained with opposite expression intensities for flower and leaf (Figure 6A). MEturquoise, MEblack, and MEgrey were the most important modules. MEturquoise and MEgrey were upregulated in the flowers, while MEblack was upregulated in the leaf.

The top 100 highly connected hub genes were identified by using the degree method in the CytoHubba plugin in Cytoscape (Chin et al., 2014; Figure 6B).

The zinc finger was the most abundant family among the highly connected hubs, followed by AP2 and MYB TFs (Figure 6C). However, the expression levels of these TFs were mainly high in the flower of *C. ensifolium* as compared with other tissues. There were 22 AP2 and 19 zinc finger members with higher expression in flowers of three species as compared with leaf tissues. The bHLHs were only four with upregulated expressions in all the tissues as compared with other families. The MYBs were comparatively upregulated in *C. ensifolium* flowers and leaf, while their expression were mainly downregulated in other species (Figure 6C).

### Flower and Leaf Specific TFs

**FIGURE 7:**
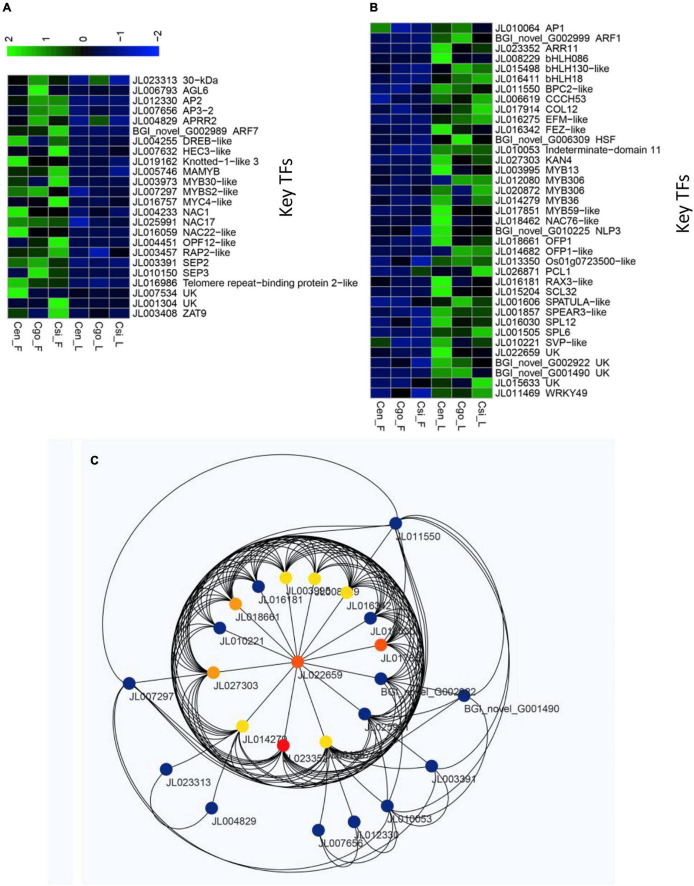
Sets of highly differential TFs among flowers and leaves of the three orchid species, including upregulated only in the flowers **(A)** and upregulated only in the leaves **(B)**; and the network formed by the flower and leaf specific TFs **(C)**.

We identified a number of TFs that upregulated either in the flowers of three species or leaves (Supplementary Table 3). There were 24 TFs that showed high expression in the flowers of three species, while they were downregulated in all the leaf samples (Figure 7A). These included the famous floral regulators, such as AP2-DREB-like, AP3-2, SEP2, SEP3, AGL6, ARF7, HEC3-like, Knotted-1-like3, and MYB TFs.

A total of 37 TFs were expressed only in the leaves of three species, while they were downregulated in the flowers (Figure 7B). These included, 5 MYB TFs, 3 bHLHs, ARF1, COL12, AP1, and 2 SPLs, which are thought to play important roles in the unique flowering pattern of orchid species.

A sub-network was constructed for the 61 TFs that were strictly specific to flower (24) and leaf (37). Through this network, we also identified important hub TFs (Figure 7C).

## Discussion

*Cymbidium sinense*, *C. ensifolium*, and *C. goeringii* are the most important commercial orchid species, contributing a major source of revenue in the orchid industry owing to their remarkable floral shapes. However, these orchids take 2–3 years to complete their vegetative phase for flowering. Therefore, recent strides have been directed to regulate flowering time in orchids, although nothing is achieved yet. Therefore, any development on quick flowering or narrow vegetative phase can be a great opportunity to induce flowering time regulation in the precious orchids. Luckily, the protocorms of the three orchid species showed abnormal flowering pattern in the controlled environment. Unlike the usual protocorm growth pattern, vegetative-to-reproductive growth cycle, some protocorms skipped the vegetative growth and produced flowers without leaves. This surprising abnormality led to perform a *de novo* transcriptome analysis to isolate commonly expressed genes in three species. Specifically, we concentrated on the TFs that regulate the expression of genes to drive abnormal changes.

Leaf and flower buds are shown to have similar chilling requirements but different requirements of heat, which may cause early flower bud bloom before leaf unfolding (Guo et al., 2014). Abnormal growth changes are mainly induced by physical means, such as irradiation and wounding, by hormonal changes and by affliction of external agents, such as bacteria, viruses, and fungi (Raghavan, 2000). Ectopic expression of *Brassica SHOOT MERISTEMLESS* genes caused abnormal phenotypes in Arabidopsis, which are supposed to be caused by differential levels of endogenous hormones (Elhiti and Stasolla, 2012). However, no particular study is available to reveal that how the reproductive cycle starts by skipping vegetative growth. Genetic changes are supposed to play major role to induce such changes. We have recently identified a number of TFs regulating flowering in the orchids (Ahmad et al., 2020, 2021a, 2022a). The current study identified 33,499 unigenes from six tissues of three orchid species. The DEGs were used as reference to mine 1,373 TFs (Supplementary Table 2), which were divided into 40 families. A number of TFs are thought to play significant roles in the regulation of abnormal flower development in the orchids. Zinc finger, MYB, bHLH, and AP2 were the most abundant TF families in the transcriptome data (Figure 5A). By comparing the flowers or leaves among the three species, it can be seen that there is no uniformity in the up- and downregulation trends at the family levels (Figure 5B). Moreover, the upregulated TFs were comparatively high in *C. goeringii* flowers (Figure 5C), suggesting that abnormal flowering might be driven by individual TFs.

**FIGURE 8:**
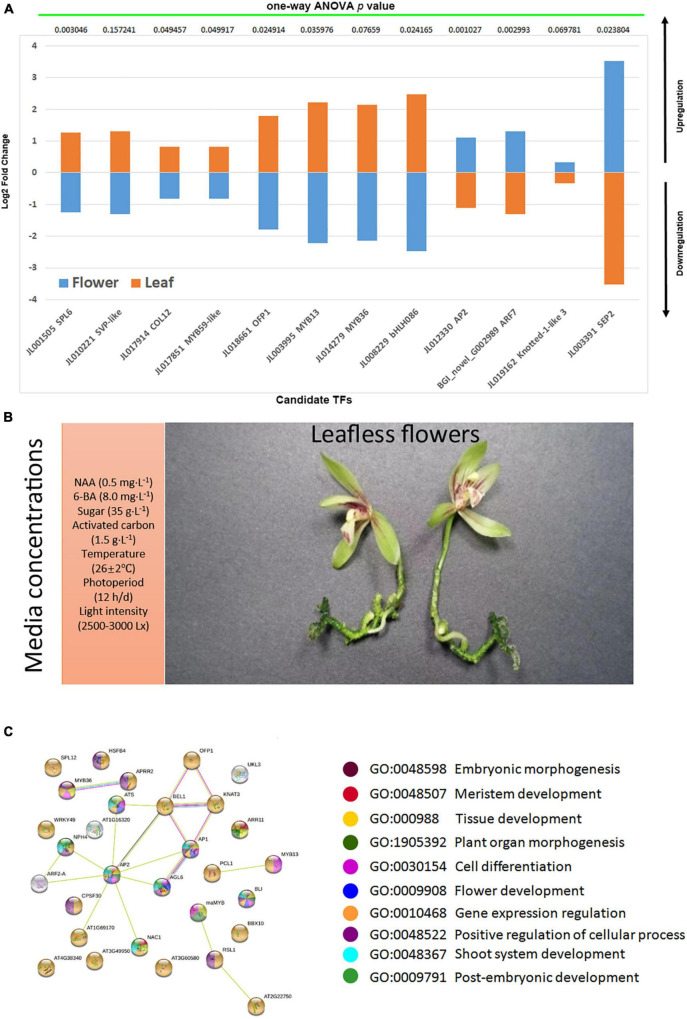
The validation of selected candidate TFs **(A)**; the abnormal flowering along with media compositions **(B)**; the string-based interaction network for the regulation of flowering **(C)**, the ball colors represent biological process enrichment.

Individual TF mining was planned to isolate only those TFs which showed consistently up- or downregulation in either the flowers of three species or leaves (Figures 7A,B). A total of 61 potent TFs were isolated (Figures 7A,B). This group contained MADS-box TFs, MYBs, bHLHs, zinc fingers, AP2, and SVP TFs with admitted role in flowering regulation in orchids (Teo et al., 2019; Valoroso et al., 2019; Ahmad et al., 2021b, 2022a, 2022b; Chen et al., 2021; Yang et al., 2021). Among the strictly flower or leaf specific TFs, 24 were upregulated in the flowers of three species (Figure 7A), while 37 TFs were downregulated in the flowers (Figure 7B). From the WGCNA modules and manual mining, we specified 12 potential TFs (*SPL6*, *SVP*-like, *COL12*, *MYB59*-like, *OFP1*, *MYB13*, *MYB36*, *bHLH086*, *AP2*, *ARF7*, *KNOX1*-like, and *SEP2*) that may play key roles in the regulation of abnormal flowering in the orchid species (Figure 8A). They were verified through nucleotide blast (Supplementary Table 3) and one-way ANOVA.

*SHORT VEGETATIVE PHASE (SVP)* is known as a flowering time regulator that has been suggested to play a role in continuous flowering in *Arundina graminifolia* (Ahmad et al., 2021b, 2022b). It regulates TCP TFs during bud bread (Singh et al., 2019). *SVP2* in *C. goeringii* interacts with *CgSOC1* and *CgAP1* to regulate flower development as a function of MADS-box TF (Yang et al., 2019). AP1 can act as a hub between flower induction proteins, such as SVP and SOC1, and the proteins of floral organ identity (Honma and Goto, 2001). In the photoperiod and temperature dependent pathways, *SVP* represses *FT* by interacting with *FLM* and *FLC*, thereby regulating flowering (Fujiwara et al., 2008; Gregis et al., 2009; Tao et al., 2012). It also involves hormonal pathways for ABA and GA during the bud break (Singh et al., 2018). *SQUAMOSA PROMOTER BINDING PROTEIN-LIKE (SPL*) TFs play roles in the regulation of flowering time, pollen development, and phase transition (Huijser and Schmid, 2011; Preston and Hileman, 2013). *CONSTANS* (*CO*) TFs regulate flowering time in the photoperiod pathway (Ordoñez-Herrera et al., 2018). The COP1/SPA E3 ubiquitin ligase in *A. thaliana* stimulates the degradation of endogenous developmental pathway regulators. CONSTANS LIKE 12 (COL12) acts as a substrate for COP1/SPA E3 ligases to control flowering time (Ordoñez-Herrera et al., 2018). Overexpressing *COL12* instigates late flowering through suppressing the expression of *FT*. Moreover, it also suppresses flowering by suppressing the function of CO protein (Andrés and Coupland, 2012; Xu et al., 2016; Ordoñez-Herrera et al., 2018).

*MYB59* is an R2R3-type TF that mainly involves cell cycle progression in Arabidopsis (Mu et al., 2009). *MYB13* functions as a transcriptional regulator of the genes involving auxin responses (Qian et al., 2020). In Arabidopsis root, *MYB36* controls the transition of cells from proliferation to differentiation (Liberman et al., 2015). *AUXIN RESPONSE FACTOR (ARF7*) is a major TF in the phototropic responses downstream of light and auxin signaling (Han and Hwang, 2018). Ovate Family Protein 1 (*OFP1*) regulates plant growth and development. It interacts with *Arabidopsis Thaliana* Homeobox 1 (*ATH1*) to coordinate flowering time and flower development (Zhang et al., 2018).

The *KNOX* genes play dual roles, regulating the vegetative and reproductive development. The expression timing of *KNOX* is crucial to establish a diverse range of floral morphologies (Rudall et al., 2013; Semiarti et al., 2014). In our case, *KNOX1* was upregulated in the flowers of three species (Figure 8A), suggesting a role for abnormal flowering. Along with this, ARF7, AP2, and SEP2 were also upregulated. However, the other MADS-box TFs, MYBs, and bHLH were downregulated (Figure 8A). This suggests that abnormal changes also suppressed a number of key floral regulators and integrators, following a different program to skip vegetative phase for rapid flowering. However, extensive functional studies are necessary to unravel the details of this unique flowering pattern.

## Conclusion

With the above study in mind, a certain ratio of growth media components elicited an interesting flowering phenotype (Figure 8B), wherein vegetative phase was skipped to allow a rapid reproductive stage. The *C. sinense* took about 180 days to flower, and the *C. ensifolium* and *C. goeringii* both took 90–120 days to flower. The TF mining elucidated important hubs that may coordinate genes regulating the vegetative development, phase transition, and the fast reproductive development. *KNOX1* along with *MYB36* and *OFP1* could be the key TFs that can drive floral integrators for flowering time regulation. The hub TFs possess important biological processes (Figure 8C) that may contribute to such strange phenotypes. These outcomes act as a hypothetical substrate to build the experimental setup for inducing rapid flowering in orchids with short vegetative phase.

## Data Availability Statement

The transcriptome data described in this article was submitted to The National Genomics Data Center (NGDC, https://ngdc.cncb.ac.cn) under accession number: PRJCA009885.

## Author Contributions

SA: conceptualization and writing the original draft. JC and GC: data curation and software. JH and XS: investigation. YH: software. YL: editing the manuscript. ST: data curation. YZ: visualization, investigation, and editing the manuscript. KZ: data curation and conceptualization. SL: software and editing the manuscript. ZL: supervision, conceptualization, and funding acquisition. DP: supervision, conceptualization, funding acquisition, and writing, reviewing, and editing the manuscript. All authors contributed to the article and approved the submitted version.

## Conflict of Interest

The authors declare that the research was conducted in the absence of any commercial or financial relationships that could be construed as a potential conflict of interest.

## Publisher’s Note

All claims expressed in this article are solely those of the authors and do not necessarily represent those of their affiliated organizations, or those of the publisher, the editors and the reviewers. Any product that may be evaluated in this article, or claim that may be made by its manufacturer, is not guaranteed or endorsed by the publisher.

## Footnotes

1
http://deweylab.biostat.wisc.edu/rsem/rsem-calculate-expression.html

## Abbreviations

AP: APETALA
ARF: auxin response factor
ATH1: Arabidopsis thaliana Homeobox 1
BHLH: basic helix–loop–helix
CO: CONSTANS
COL12: CONSTANS LIKE12
DOH1: Dendrobium Orchid Homeobox1
FT: Flower locus T
FUL: FRUITFUL
KNOX1: KNOTTED1-like homeobox
LFY: LEAFY
MYB: v-myb avian myeloblastosis viral oncogene homolog
OFP1: Ovate Family Protein1
PLB: protocorm like body
POH1: Phalaenopsis Orchid Homeobox1
SAM: Shoot apical meristem
SEP: SEPALLATA
SOC: SUPPRESSION OF OVEREXPRESSION OF CONSTANS
SPL: SQUAMOSA PROMOTER BINDING LIKE
SVP: SHORT VEGETATIVE PHASE
TCP: Teosinte branched1/Cycloidea/Proliferating cell factor
TOM: topology matrix
WGCNA: weighted gene coexpression network analysis.
